# Facile fabrication of water-dispersible nanocomposites based on hexa-*peri*-hexabenzocoronene and Fe_3_O_4_ for dual mode imaging (fluorescent/MR) and drug delivery

**DOI:** 10.1039/c8ra08425d

**Published:** 2018-12-05

**Authors:** Meng-Meng Song, Hui-Hui Xiang, Meng-Yu Fei, Da-Peng Lu, Tong-Cui Jiang, Yong-Qiang Yu, Rui Liu, Yu-Xian Shen

**Affiliations:** School of Basic Medical Sciences, Anhui Medical University 81 Meishan Road Hefei Anhui PR China liurui@ahmu.edu.cn shenyx@ahmu.edu.cn +86-551-65161138 +86-551-65113776; Biopharmaceutical Research Institute, Anhui Medical University 81 Meishan Road Hefei Anhui PR China; The First Affiliated Hospital, Anhui Medical University 218 Jixi Road Hefei Anhui PR China; School of Pharmacy, Anhui Medical University 81 Meishan Road Hefei Anhui PR China

## Abstract

The facile fabrication of multifunctional nanocomposites (Fe_3_O_4_/HBC@F127) consisting of superparamagnetic Fe_3_O_4_ nanoparticles and fluorescent organic hexa-*peri*-hexabenzocoronene (HBC) molecules incorporated in block copolymer diacylphospholipid–polyethyleneglycol F127 have been demonstrated for dual mode imaging (fluorescent/MR) and drug delivery. The obtained nanocomposites were water-dispersible, stable and biocompatible, as confirmed by dynamic light scattering (DLS) and 3-(4,5-dimethylthiazol-2-yl)-2,5-diphenyltetrazolium bromide (MTT) assay. Relativity measurements showed a *T*_2_ relaxivity (*r*_2_) of 214.61 mM^−1^ s^−1^, which may be used as *T*_2_-weighted MR imaging agents. *In vitro* imaging studies indicated that the nanocomposites had good MR and fluorescence imaging effects with low cytotoxicity. Besides, the developed nanocomposites could also be applied as drug delivery vehicles. Doxorubicin (DOX) loaded Fe_3_O_4_/HBC@F127 nanocomposites significantly inhibited the growth of human hepatoma cells (HepG2). These findings suggested that the facile synthesized multifunctional nanocomposites may be used as a platform for dual mode imaging (both MR and fluorescence) and drug delivery.

## Abstract 2

Water-dispersible, stable and biocompatible dandelion-like Fe_3_O_4_/HBC@F127 nanocomposites were facilely developed for dual mode imaging (fluorescent/MR) and drug delivery.

## Graphical abstract

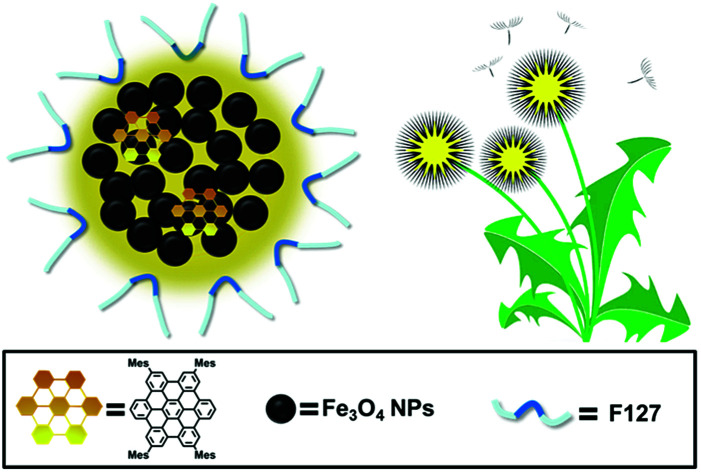

## 1 Introduction

Fluorescence imaging plays an important role in bio-life science because of the emission of the probes after excitation can be visualized by the naked eye or at high resolution with optical microscopy.^1,2^ An ideal fluorescent probe for bio-imaging should be bright, biocompatible, water-soluble, and stable against photo bleaching.^3^ It is well known that traditional organic dyes are favored for routine bio-imaging and especially for applications requiring very accurate quantification.^4,5^ Nevertheless, the problem of hydrophobicity has severely restrained their wide spread applications to biological systems.^6^ For example, hexa-*peri*-hexabenzocoronene (HBC) and its derivatives, as nanographene segments, have attracted considerable attention because of their applications in field-effect transistors and photoconductive devices.^7–9^ However, their applications in biosystems are limited. Multicolor graphene quantum dots (GQDs) with a uniform size of 60 nm diameter and 2–3 nm thickness were synthesized by using a substituted HBC as the carbon source for bio-imaging.^10^ Yin *et al.* reported a novel water-soluble HBC derivative with negatively charged side arms that could self-assemble into micro- and nanofibers for bioprobing.^11^ Considering the planar structure of HBC, it could aggregate and self-assemble into different morphologies in aqueous solution.^12,13^

Although fluorescence imaging shows high sensitivity at subcellular levels and is suitable for quantifying, it still has limitations in examining deep tissue *in vivo*.^14^ Therefore, the combined nanoprobe, for example, a nanoprobe both for fluorescence imaging and MR imaging have great significance due to their complementary capabilities.^15^ Among various contrast agents, superparamagnetic Fe_3_O_4_ nanoparticles have been widely developed as negative contrast agents that can shorten the *T*_2_ relaxation time of water protons, resulting in enhanced imaging contrast and sensitivity.^16,17^

One important issue related to the applications of HBC molecules and Fe_3_O_4_ nanoparticles for fluorescence imaging and *T*_2_ weighted MR imaging is how to render the hydrophobic molecules and Fe_3_O_4_ particles with good water-dispersity, biocompatibility and long blood circulation time. The formation of non-cytotoxic copolymer micelles has been proved to be an effective strategy to make the particles meet the above requirements. Polyethylene glycol (PEG) based Pluronic triblock co-polymers are amphiphilic and composed of a hydrophobic central segment of poly(propylene oxide) (PPO) and two hydrophilic segments of poly(ethylene oxide) (PEO), which are widely used as a coating/stabilizing agent for the surface modification of nanoparticles for various biomedical applications.^18–21^ The amphiphilic block copolymer diacylphospholipid–polyethyleneglycol (F127) can conveniently encapsulate hydrophobic molecules with simple steps and the obtained F127-coated nanocomposites is a kind of ideal drug delivery system with good biocompatibility and prolonged blood circulation time. Hydrophobic drug can also be loaded by physical encapsulation due to its self-assembly characteristic.^22–24^ Take these factors into consideration, it is believed that the combination of HBC, Fe_3_O_4_ nanoparticles and polymer could be a new method for the design of multifunctional nanocomposites for dual mode imaging (fluorescent/MR) and drug delivery.

Herein, we developed a novel water-dispersible Fe_3_O_4_/HBC@F127 nanocomposite by simply incorporating two hydrophobic functional particles (HBC molecules and Fe_3_O_4_ nanoparticles) in a biodegradable polymeric micelle of F127. The formed particle showed good MR and fluorescence imaging effects with low cytotoxicity. It also can be applied as a drug delivery vehicle by loading Doxorubicin (DOX) to inhibit the growth of HepG2 cells. The Fe_3_O_4_/HBC@F127 multifunctional nanocomposites could be a potential platform for dual mode imaging (fluorescent/MR) and drug delivery.

## 2 Experimental

### 2.1 Materials

Pluronic F127 was purchased from Aldrich (St. Louis, USA). DOX·HCl was purchased from Beijing Huafeng United Technology Co. Ltd. (Beijing, China). 3-(4,5-Dimethylthiazol-2-yl)-2,5-diphenyltetrazolium bromide (MTT) and 4,6-diamino-2-phenyl indole (DAPI) was acquired from Shanghai Sangon Biological Engineering Technology & Services Co., Ltd (China). Dulbecco's Modified Eagle Medium (DMEM), fetal bovine serum (FBS), penicillin, and streptomycin were from Hangzhou Jinuo Biomedical Technology (Hangzhou, China). Other chemicals were purchased from Sinopharm Chemical Reagent Co., Ltd. (Shanghai, China).

### 2.2 Synthesis of Fe_3_O_4_/HBC@F127 nanocomposites

Tetramesityl HBC molecules was synthesized according to the reported method.^25^ Hydrophobic Fe_3_O_4_ nanoparticles were synthesized as previously reported.^26^ To synthesize Fe_3_O_4_/HBC@F127 nanocomposites, F127 (5 mg), HBC (5 mg) and Fe_3_O_4_ (5 mg) nanoparticles were dissolved in 0.5 mL THF and then added dropwise into 30 mL H_2_O and then kept stirring for 30 min. The resulting solution was then dialyzed in pure water for 3 days. Finally, the resulting suspension was filtered through a 0.22 μm membrane before use. DOX (5 mg) was also added in the first step to obtain DOX loaded Fe_3_O_4_/HBC@F127 nanocomposites.

### 2.3 Characterizations

Transmission electron micrographs (TEM) were taken by TEM (JEOL JEM-2100F) for particle size determination. Infrared spectra were recorded in the range 4000–400 cm^−1^ on a Fourier-transform infrared spectrometer (FT-IR, Bomen Hartmann and Braun, MB series). Dynamic light scattering (DLS) measurement was determined by Zetasizer (Nano series, Malvern Instruments). The magnetic measurement was carried out using vibrating sample magnetometer (VSM, MPMS SQUIDM, USA). The absorption and emission spectra was analyzed by using a UV-vis spectroscopy (Specord 205, Jena, Germany) and fluorescence spectroscopy (Cary Eclipse, Varian, USA). The Fe concentration of the particles was determined by inductively coupled plasma analyses (ICP, Agilent 730 ICP-OES, USA). The *T*_2_ relaxation times of the particle suspension with different Fe concentrations in water were measured by a 3.0-T GE Signa HDX MRI scanner (GE, Milwaukee, USA) by using a head coil. *T*_2_-weighted images were obtained from a 4.0 mm-thick section using a 60 mm × 48 mm field of view (FOV), repetition time (TR) = 3000 ms, echo time (TE) = 40, 60, 80, 100 and 120 ms and reconstructed using a 320 × 320 image matrix. The relaxivity (*r*_2_) was calculated by a linear fitting of the inverse relaxation time as a function of the Fe concentration.

### 2.4 Cell culture, cytotoxicity, and cellular uptake

Human hepatoma cells (HepG2) were obtained from American Type Culture Collection (ATCC, Manassas, VA, USA) and cultured in DMEM medium. Each cell culture medium was supplemented with 10% inactivated fetal bovine serum (FBS), 100 mg mL^−1^ streptomycin, and 100 U mL^−1^ penicillin at 37 °C under 5% CO_2_. For cytotoxicity study, the cells were seeded on a 96-well plate at a density of 1 × 10^4^ cells per well with DMEM medium containing 10% FBS. After 24 h, the culture medium was replaced with 100 μL of medium containing 0–400 μg mL^−1^ of Fe_3_O_4_/HBC@F127 nanocomposites. The cytotoxicity was evaluated by determining the cell viability after incubation for 24 h. The number of viable cells was determined by MTT assay according to the reported method.^27^

For cellular uptake study, the cells (5 × 10^5^) were pre-grown in 6-well culture plates and then Fe_3_O_4_/HBC@F127 nanocomposites were added at a concentration of 0–40 μg mL^−1^ in the same medium and then incubated separately for 1 h, 2 h and 24 h. Next, the culture medium was aspirated and the cells were washed three times with 2 mL of PBS containing 2% FBS. The cells were detached by 1× trypsin and centrifuged at 1200 rpm for 5 min. The media was then removed by aspiration. The cells were re-suspended in 2 mL of PBS and 1 × 10^4^ cell accounts were immediately analyzed using a flow cytometer (Beckman Coulter, California, USA). The collected cells were microwave digested and then the cellular uptake Fe was analyzed by ICP.

For cell imaging, the cells (5 × 10^4^) were seeded into a 24-well culture plate containing one glass coverslip per well and incubated for 24 h. Next, the medium was removed and 0.5 mL of DMEM media containing Fe_3_O_4_/HBC@F127 nanocomposites was added into each well and incubated at 37 °C for 24 h. The coverslips with cells were then placed in empty wells, treated with 1 mL of 4% formaldehyde in PBS, and allowed to sit at room temperature for 30 min. After washing with PBS for three times, the cells were treated with 1 mL of Triton X-100 and incubated for 10 min. Then the cells were washed three times with PBS and then incubated at 37 °C with 0.2 mL of DAPI for 10 min. The cells were analyzed using a confocal laser scanning microscope (CLSM, Zeiss, LSM 880, Germany).

For MR imaging, after the co-incubation with Fe_3_O_4_/HBC@F127 nanocomposites, the cells were washed thoroughly, then dispersed and suspended by 2 mL 0.5% agarose gel in a 5 mL EP tube. MR imaging was performed on a 3.0-T GE Signa HDX MRI scanner (GE, Milwaukee, WI, USA) using a *T*_2_-weighted Fast Spin Echo sequence (TR = 3000 ms, TE = 100 ms, slice thickness of 4 mm).

## 3 Results

### 3.1 Fabrication and characterizations of Fe_3_O_4_/HBC@F127 nanocomposites

**Scheme 1:**
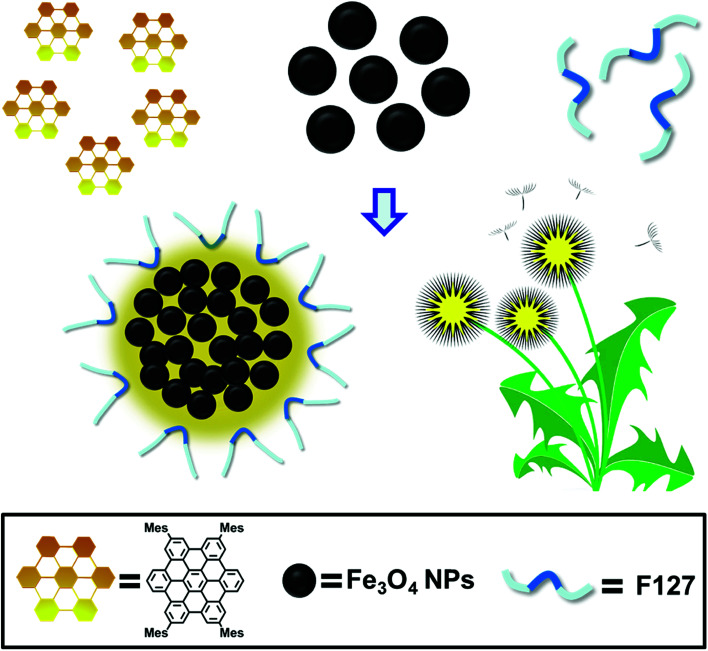
Schematic illustration of the formation of dandelion-like Fe_3_O_4_/HBC@F127 nanocomposites.

The schematic illustration of the simply formation of Fe_3_O_4_/HBC@F127 nanocomposites was shown in Scheme 1. Hydrophobic oleic acid-coated Fe_3_O_4_ nanoparticles and HBC molecules were dissolved in THF with F127, and then the mixed solution was added into deionized H_2_O to form the dandelion-like micelles. After completely dialysis, the micelles containing Fe_3_O_4_ nanoparticles and HBC molecules were formed, which abbreviated as Fe_3_O_4_/HBC@F127. Pluronic F127 is selected to encapsulate HBC and Fe_3_O_4_ nanoparticles due to its amphiphilic nature and excellent biocompatibility as a copolymer consisting poly(ethylene oxide)–poly(propylene oxide)–poly(ethylene oxide) blocks, PEO100–PPO65–PEO100. During the formation of nanocomposites, the hydrophobic PPO is more likely wrapped inside the particles with hydrophobic HBC and Fe_3_O_4_ to prevent aggregation, protein adsorption, and recognition by the reticuloendothelial system (RES).^28^

**Fig. 1:**
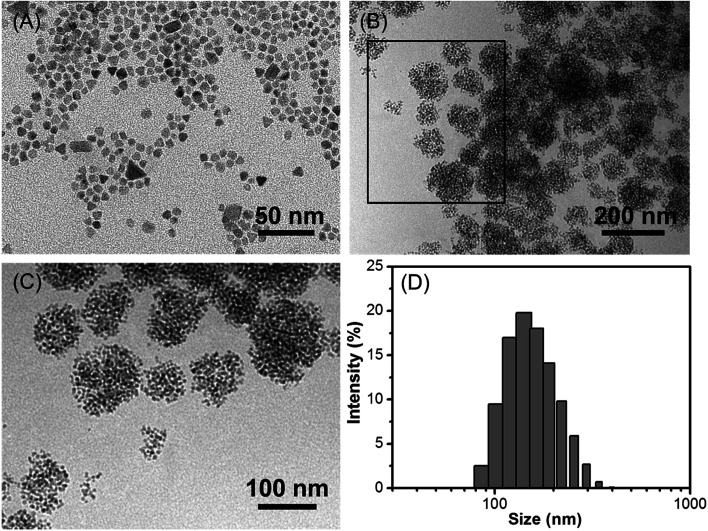
Morphology characterizations of Fe_3_O_4_/HBC@F127 nanocomposites. (A) TEM micrographs of Fe_3_O_4_ nanoparticles, (B) Fe_3_O_4_/HBC@F127 nanocomposites, (C) a magnified view of the rectangular area of (B) and (D) size distribution of Fe_3_O_4_/HBC@F127 nanocomposites by DLS.

The morphology of Fe_3_O_4_/HBC@F127 nanocomposites was studied by TEM. Fe_3_O_4_ nanoparticles were obtained as colloidal particles dispersed in common nonpolar or weakly polar solvents, such as hexane, tetrahydrofuran, chloroform, *etc.* A representative TEM image shows that Fe_3_O_4_ nanoparticles modified by oleic acid in hexane are highly monodisperse, with an average diameter of about 10 nm (Fig. 1A). After entrapped with HBC in block copolymer F127, some hydrophobic Fe_3_O_4_ nanoparticles are aggregated in the inner space of the F127 micelle due to the hydrophobic interaction (Fig. 1B). Simultaneously, HBC has a strong tendency to aggregate through π–π stacking when entrapped into block copolymer F127 due to its planar core. The fabricated Fe_3_O_4_/HBC@F127 nanocomposites have a nearly spherical morphology. It can be seen clearly that each particles are made of many Fe_3_O_4_ nanoparticles in the magnified TEM image (Fig. 1C). The mean hydrodynamic diameter was in the range of 100–300 nm measured in deionized H_2_O. Besides, Fe_3_O_4_/HBC@F127 nanocomposites are stable in deionized water for a period of 3 months without aggregation, which is desirable for application in biomedicine.

**Fig. 2:**
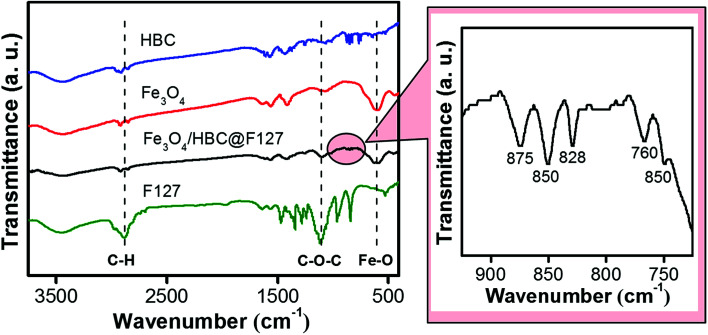
FT-IR spectra of HBC molecules, Fe_3_O_4_ nanoparticles, Fe_3_O_4_/HBC@F127 nanocomposites and F127.

Fourier-transform infrared (FT-IR) spectroscopy was performed to investigate the effective compound of HBC molecules, Fe_3_O_4_ nanoparticles and F127. As shown in Fig. 2, the absorption band at around 597 cm^−1^ of Fe_3_O_4_/HBC@F127 nanocomposites corresponding to the Fe–O bond in Fe_3_O_4_ conforms the presence of Fe_3_O_4_ nanoparticles.^29^ Even though the absorption bands of HBC at 875, 850, 828, 766 and 750 cm^−1^ are suppressed in Fe_3_O_4_/HBC@F127 nanocomposites (circled in Fig. 2) due to the strong background of Fe_3_O_4_ nanoparticles, they still can be clearly figured out in the magnified spectrum. Moreover, the characteristic absorption bands of F127 at 2886.4 cm^−1^ (C–H asymmetric stretching) and 1111.4 cm^−1^ (C–O–C stretching) also appear in the FT-IR spectrum, which indicate the effective coating onto the surface of Fe_3_O_4_ nanoparticles.^30^ The characteristic band of F127 at 2882 cm^−1^ (C–H asymmetric stretching) is overlapped with the peaks at 2915 cm^−1^ and 2855 cm^−1^ in FT-IR spectrum of Fe_3_O_4_/HBC@F127 nanocomposites, which is hard to identify.

**Fig. 3:**
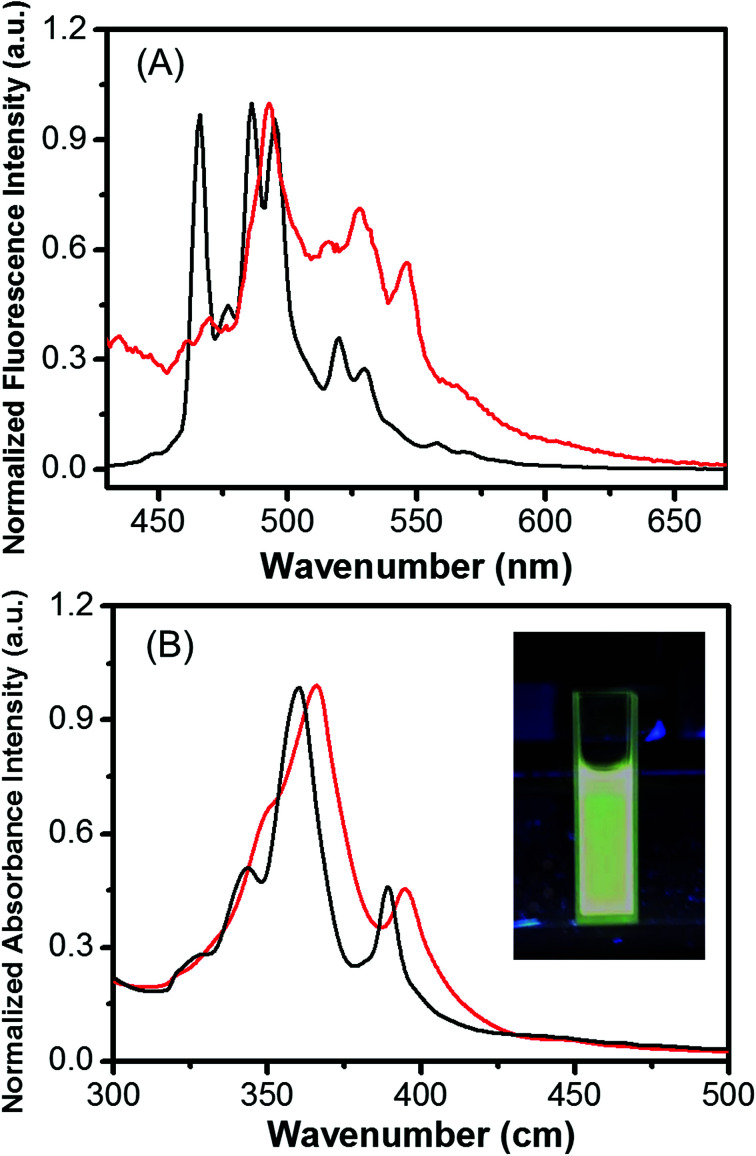
UV-vis absorption spectra (A) and fluorescence spectra (B) for HBC in THF and Fe_3_O_4_/HBC@F127 nanocomposites in deionized H_2_O (black line-HBC in THF, red line-Fe_3_O_4_/HBC@F127 nanocomposites in deionized H_2_O).

The optical properties of HBC in THF and Fe_3_O_4_/HBC@F127 nanocomposites dispersed in deionized H_2_O were investigated by UV-vis spectrometer and fluorescence spectrometer. The absorption and emission intensity are both normalized in Fig. 3. Both the absorption and emission spectra of Fe_3_O_4_/HBC@F127 nanocomposites in deionized H_2_O are similar with those of HBC in THF. The UV-vis absorption spectra showed a strong absorption at around 360 nm, which was selected as the excitation wavelength (Fig. 3A). The emission spectrum of Fe_3_O_4_/HBC@F127 nanocomposites showed multiple emission bands with maximum peaks at 468, 506 and 586 nm (Fig. 3B). As shown in the inset in Fig. 3B, the Fe_3_O_4_/HBC@F127 nanocomposites also present an intense greenish photoluminescence under UV lamp irradiation.

**Fig. 4:**
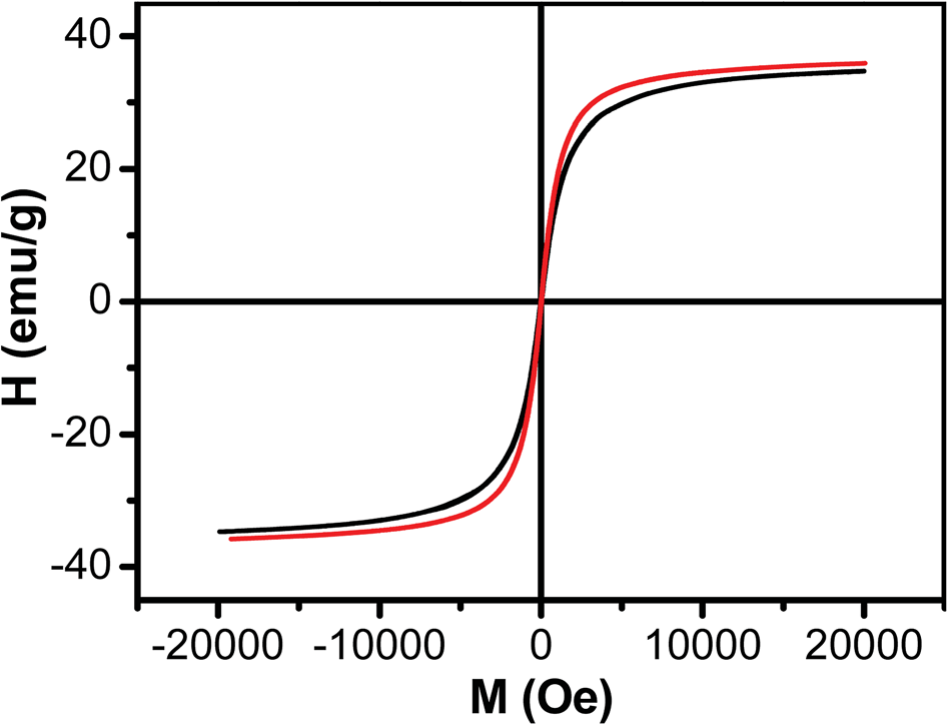
Magnetization curve as a function of field for Fe_3_O_4_ nanoparticles (red line) and Fe_3_O_4_/HBC@F127 nanocomposites (black line) at 300 K.

The magnetic property of Fe_3_O_4_ nanoparticles and Fe_3_O_4_/HBC@F127 nanocomposites was evaluated by field-dependent magnetization measurements at 300 K (Fig. 4). The lack of hysteresis indicates the superparamagnetic nature of the Fe_3_O_4_ nanoparticles. The saturated magnetization of Fe_3_O_4_ nanoparticles was 35.57 emu g^−1^ when the applied magnetic field reaches 20 kOe. The saturated magnetization decreased slightly (34.67 emu g^−1^) when Fe_3_O_4_ nanoparticles formed Fe_3_O_4_/HBC@F127 nanocomposites, which was attributed to the decrease of Fe_3_O_4_ content of the nanocomposites. The data around zero field showed the coercivity of Fe_3_O_4_/HBC@F127 nanocomposites was 26 Oe, which demonstrated that the Fe_3_O_4_/HBC@F127 nanocomposites were superparamagnetic without hysteresis.

### 3.2 MR relaxivity measurements

**Fig. 5:**
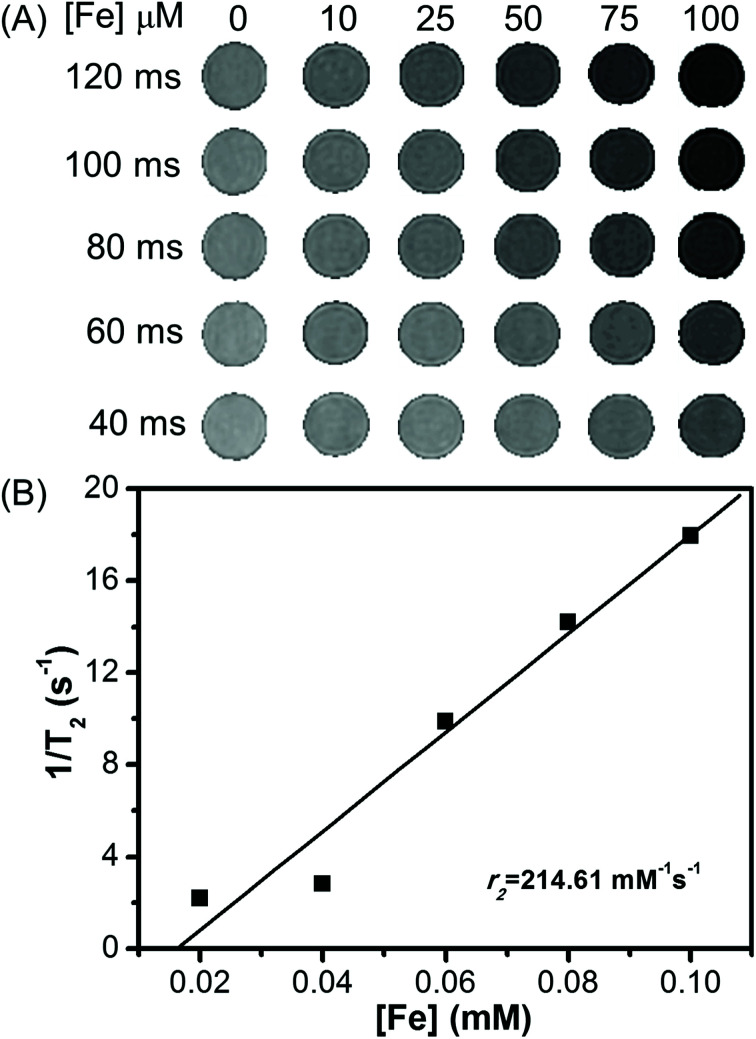
Magnetic resonance imaging properties of Fe_3_O_4_/HBC@F127 nanocomposites: (A) *T*_2_-weighted MR images of Fe_3_O_4_/HBC@F127 nanocomposites in PBS solution at 3.0 T MRI system at various iron concentrations for different values of echo time (TE, TR = 3000 ms). (B) *T*_2_ relaxivity plot of aqueous suspension of Fe_3_O_4_/HBC@F127 nanoparticles *vs.* iron concentration (data as values obtained from curve fitting).

The MR imaging properties of Fe_3_O_4_/HBC@F127 nanocomposites were evaluated by measuring the transverse relaxation time *T*_2_ on a 3.0 T scanner. Their efficiency as contrast agents is determined by calculating the transverse relaxation rate from a linear fit of the inverse relaxation times as a function of the iron concentration. The transverse relaxation time *T*_2_ of water was reduced by Fe_3_O_4_/HBC@F127 nanocomposites relative to the control PBS. As shown in Fig. 5A, we observed that the *T*_2_-weighted MR signal intensity decreased with the increase of Fe_3_O_4_/HBC@F127 nanocomposites concentration (measured in μM Fe) at different TE values (TR = 3000 ms). As expected, the relaxation rate *r*_2_ is equal to 1/*T*_2_, which is linearly proportional to Fe concentration (Fig. 5B). *T*_2_ relaxivities *r*_2_ for our Fe_3_O_4_/HBC@F127 nanocomposites is 214.61 mM^−1^ s^−1^, which is higher than that of Feridex (*r*_2_ = 108.2 mM^−1^ s^−1^).^31^

### 3.3 *In vitro* imaging studies

**Fig. 6:**
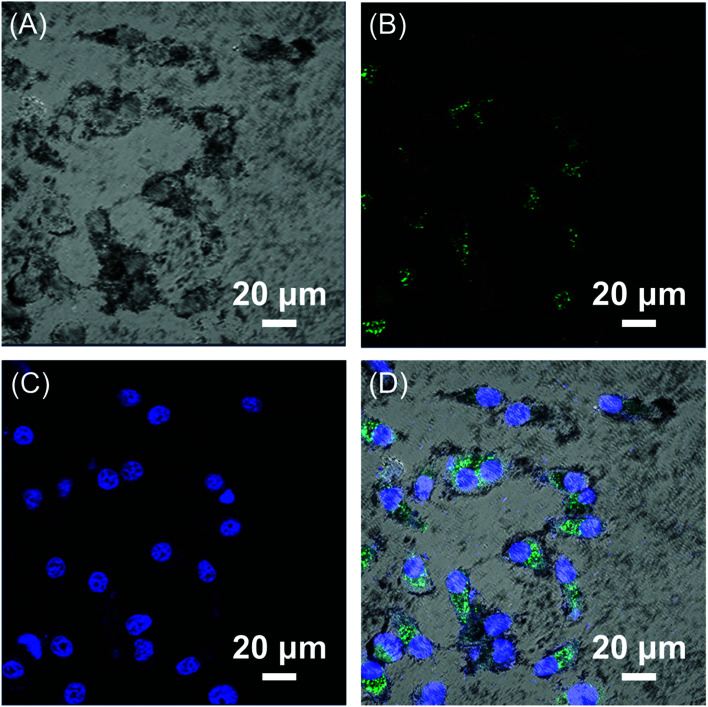
CLSM images of HepG2 cells exposed to 10 μg mL^−1^ Fe_3_O_4_/HBC@F127 nanocomposites for 24 h. (A) Bright field, (B) Fe_3_O_4_/HBC@F127 nanocomposites, (C) DAPI, and (D) merged image of (A)–(C).

The encapsulated HBC molecules enabled confocal microscopic tracking of the nanocomposites after internalized by cells. In order to observe the cell imaging effect, HepG2 cells were treated with Fe_3_O_4_/HBC@F127 nanocomposites at a concentration of 10 μg mL^−1^ for 24 h. The confocal microscopic images show that large amount of green particles are dispersed in the cytoplasm, which proves the cellular uptake of the nanocomposites (Fig. 6).

**Fig. 7:**
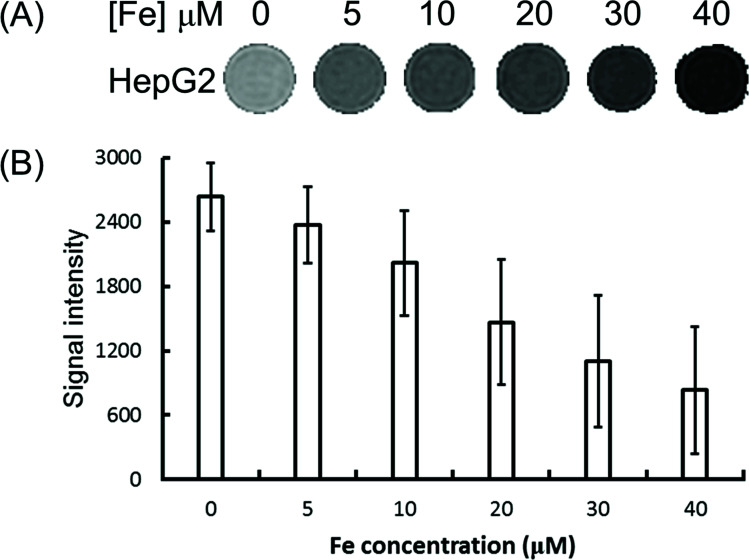
*T*_2_-weighted MR images. HepG2 cells were treated with Fe_3_O_4_/HBC@F127 nanocomposites at various Fe concentrations for 24 h. The images were collected by MR (TR = 3000 ms, TE = 100 ms) (A) and the signal intensity was analyzed (B).

To explore the feasibility of the Fe_3_O_4_/HBC@F127 nanocomposites used as contrast agents for *T*_2_-weighed MR imaging of cancer cells, HepG2 cells were incubated with Fe_3_O_4_/HBC@F127 nanocomposites at various iron concentrations for 24 h and then MR imaging investigation was performed. It can be seen from the *T*_2_-weighted MR phantom images that cells incubated with Fe_3_O_4_/HBC@F127 nanocomposites show a significantly darker signal compared with that in control group (Fig. 7A), which indicates the negative contrast enhancement becomes stronger as Fe concentration increases. The quantitative analysis of the MR signal intensity was also carried by plotting the MR signal intensity of the cells as function of Fe concentration (Fig. 7B). The MR signal intensity of cells treated by Fe_3_O_4_/HBC@F127 nanocomposites is much lower than that of the control cells treated with PBS, which suggests that the nanocomposites can specifically affect the MR signal.

### 3.4 Effects of DOX loaded Fe_3_O_4_/HBC@F127on cell viability

**Fig. 8:**
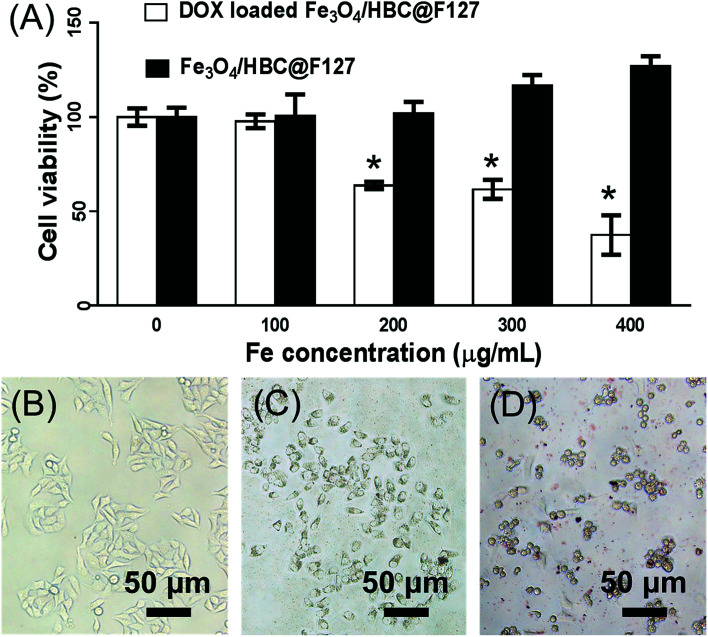
Effects of DOX loaded Fe_3_O_4_/HBC@F127 on cell viability. (A) The cytotoxic effects of Fe_3_O_4_/HBC@F127 and DOX loaded Fe_3_O_4_/HBC@F127 on HepG2 cells were detected by MTT assay. *p < 0.05 compared with control group (B) control cells treated with DMEM. (C) Cells treated with Fe_3_O_4_/HBC@F127 nanoparticles. (D) Cells treated with DOX loaded Fe_3_O_4_/HBC@F127 nanocomposites at Fe concentration of 100 μg mL^−1^. The data were presented as mean ± SD, *n* = 4.

Cytotoxicity studies were carried out to determine the cell viabilities of Fe_3_O_4_/HBC@F127 nanocomposites and DOX loaded Fe_3_O_4_/HBC@F127 nanocomposites in HepG2 cells. MTT results shows that the developed Fe_3_O_4_/HBC@F127 nanocomposites are non-cytotoxic in the given concentration range (Fig. 8A). The cell viability is found no significant difference between Fe_3_O_4_/HBC@F127 nanocomposites treated group and control group even when the concentration of Fe_3_O_4_/HBC@F127 is high up to 400 μg mL^−1^. However, the cell viability exhibited significant change when exposed to DOX loaded Fe_3_O_4_/HBC@F127 nanocomposites. DOX loaded Fe_3_O_4_/HBC@F127 nanocomposites exhibits the typical concentration-dependent antiproliferative effect on HepG2 cells. There are significant differences between DOX loaded Fe_3_O_4_/HBC@F127 treated group and control group when the concentration of Fe_3_O_4_/HBC@F127 nanocomposites is larger than 200 μg mL^−1^ (the DOX loading amount is approximately 20 μg mg^−1^ Fe_3_O_4_/HBC@F127 nanocomposites). The toxicity of DOX loaded Fe_3_O_4_/HBC@F127 nanocomposites is further visualized directly by cellular imaging (Fig. 8B–D), which confirms that higher percentage of cell death as compared to the control. Cells treated with an equivalent amount of control nanocomposites (without drug) does not show any morphology change, as the cell growth is almost identical as the control cells.

### 3.5 Cellular uptake studies

**Fig. 9:**
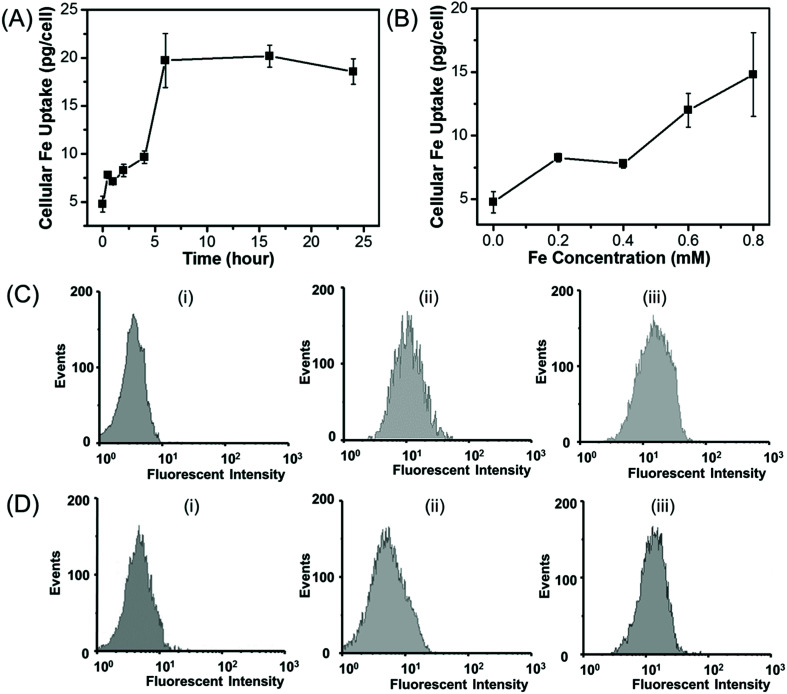
Cellular uptake of Fe_3_O_4_/HBC@F127 nanocomposites. (A and B) Fe uptake analysis by ICP at different concentrations and different time points. (C) Flow cytometry histograms of HepG2 cells incubated with Fe_3_O_4_/HBC@F127 nanocomposites for 24 h. (i) Control, (ii) 0.4 mM and (iii) 0.8 mM. (D) Flow cytometry histograms of HepG2 cells incubated with Fe_3_O_4_/HBC@F127 nanocomposites at a concentration of 0.2 mM: (i) control, (ii) 2 h and (iii) 24 h.

The amount of the cellular uptake of iron was quantitatively measured by using ICP. The cellular Fe uptake displays both concentration-dependent and time-dependent manner for HepG2 cells. As shown in Fig. 9A, when HepG2 cells are incubated with Fe_3_O_4_/HBC@F127 nanoparticles at a Fe concentration of 0.2 mM, the amount of the cellular iron increases in the first several hours and then nearly reaches a saturation point, which also suggests that the uptake of Fe_3_O_4_/HBC@F127 nanocomposites occurs within 6 hours. As shown in Fig. 9B, when HepG2 cell are incubated with Fe_3_O_4_/HBC@F127 nanocomposites for 24 h, the amount of cellular Fe grows in the tested concentration from 0 to 0.8 mM, and higher Fe concentration leads to an increased Fe uptake in the cells. Due to the fluorescence signal of HBC in Fe_3_O_4_/HBC@F127 nanocomposites, flow cytometry analysis was used to investigate the cellular uptake of Fe_3_O_4_/HBC@F127 nanocomposites in HepG2 cells (Fig. 9C and D). Compared with the control group, cells incubated with Fe_3_O_4_/HBC@F127 nanocomposites show a fluorescent shift. When the concentration increases to 0.4 mM, the fluorescent intensity does not change significantly (Fig. 9C), which is different from ICP analysis results. This can be ascribed to that the excessive nanoparticles are not able to be washed thoroughly in the cell collection process for ICP analysis as the concentration increases. The fluorescent intensity of cells incubated with Fe_3_O_4_/HBC@F127 nanocomposites for 2 h shows only a slight shift compared with control group, suggesting a weak cellular uptake efficacy. While the incubation time was prolonged to 24 h, the increased fluorescent intensity showed a better cellular uptake efficacy, which confirmed the time dependent uptake of Fe_3_O_4_/HBC@F127 nanoparticles by HepG2 cells (Fig. 9D).

## 4 Discussions

Multifunctional nanostructures have attracted wide attention, such as the combination of magnetic nanoparticles and fluorescence nanoparticles or molecules yields a bi-functional nanocomposites for fluorescence imaging and MR imaging.^32–34^ Although multifunctional nanocarriers combined with imaging and therapy has reported in many literatures, this work also has advantages. Compared with the previous publications, this work developed a trifunctional nanocomposites not only for cancer diagnosis *via* MR/fluorescence dual modal imaging but also can load drug molecules for cancer therapy. Besides, we prepared this multifunctional nanocomposites by using a facile and mild method without complex chemical reaction. Hydrophobic HBC molecules and hydrophobic Fe_3_O_4_ nanoparticles were encapsulated by using biocompatible copolymer F127 due to the hydrophobic interaction and π–π stacking interaction. The significance of this combination also lies in broadening the application of hydrophobic HBC molecules in biomedicine. On one hand, this combination increases the water dispersibility of HBC molecules *via* F127 micellar solubilization, which is a prerequisite condition for biological application; on the other hand this combination reduces the toxicity of organic HBC molecules. As is well-known, toxicity is an important issue for biomedical imaging and drug delivery applications. It has been reported that the surface modification of the nanoparticles had a significant influence on the cytotoxicity.^35^ HBC molecules aggregated and then located in the inner space of F127 micelles with a compatible shell layer, which reduce its toxicity obviously. After self-assembly, HBC aggregates also exhibited similar brilliant fluorescent properties, HepG2 cells incubated with Fe_3_O_4_/HBC@F127 nanocomposites exhibited strong intracellular green fluorescence signal.

Supermagnetic Fe_3_O_4_ nanoparticles is a kind of ideal MR imaging contrast agents due to their high transverse relaxation profiles.^36^ Usually, a high *r*_2_ relaxivity is helpful for MR imaging. Here, the ultra-small Fe_3_O_4_ nanoparticles forms aggregates in the formation of Fe_3_O_4_/HBC@F127 nanocomposites and therefore the nanocomposites have a high *r*_2_ value of 214.61 mM^−1^ s^−1^. Compared with FDA approved Fe_3_O_4_ nanoparticles-based *T*_2_ contrast agent Feridex (*r*_2_ = 108.2 mM^−1^ s^−1^),^31^ this *r*_2_ value is high enough to yield strong negative contrast signal. The MR signal of the tumor cell samples are also efficiently decreased after incubation with Fe_3_O_4_/HBC@F127 nanocomposites, which indicated that the magnetic property of the composites are not affected by the cellular physiological environment and therefore can be used as contrast agents for MR imaging. However, it has been suggested that the potential of Fe_3_O_4_/HBC@F127 nanocomposites as an *T*_2_ contrast agent should be assessed through *in vivo* experiments.

To sum up, Fe_3_O_4_/HBC@F127 nanocomposites show good fluorescence/MR imaging ability and cell inhibition effect, which ensure its potential application for the diagnosis and treatment of cancer disease.

## 5 Conclusions

Water-dispersible Fe_3_O_4_/HBC@F127 nanocomposites were developed as multifunctional MR/fluorescence imaging agents for cancer cells. *In vitro* tests indicated that the MR/fluorescence imaging could be realized with high sensitivity by the application of Fe_3_O_4_/HBC@F127 nanocomposites. Besides, Fe_3_O_4_/HBC@F127 nanocomposites could also delivery anticancer drugs to inhibit the proliferation of HepG2 cells. The fabricated Fe_3_O_4_/HBC@F127 nanocomposites with good biocompatibility are hopeful to be developed as a new multifunctional fluorescence/MR imaging agent for the diagnosis of cancer and a delivery vehicle for the treatment of cancer.

## Conflicts of interest

There are no conflicts to declare.

## Supplementary Material
